# Interleukin 7 inhibit autophagy via P53 regulated AMPK/mTOR signaling pathway in non-small cell lung cancer

**DOI:** 10.1038/s41598-022-14742-5

**Published:** 2022-07-01

**Authors:** Yunjia Zhu, Xi Jiang, Zhiying Ding, Jian Ming

**Affiliations:** 1 Department of Pathology, The Second Affiliated Hospital of Chongqing Medical University, No.74, Linjiang Road, Yuzhong Distrist, Chongqing, 400010 China; 2 Department of Pathology, General Hospital of Northern Theater Command, No.83, Wenhua Road, Shenhe District, Shenyang, 110016 Liaoning China; 3 Department of Pathology, Tianjin Third Central Hospital, No.83 Jintang Road, Hedong District, Tianjin, 300000 China

**Keywords:** Cancer, Molecular biology, Biomarkers, Molecular medicine, Oncology

## Abstract

Interleukin 7 (IL-7) has been demonstrated regulating lymphangiogenesis, apoptosis, and proliferation. Whether IL-7 induce or inhibit autophagy in non-small cell lung cancer (NSCLC) are unknown. In this study, Western blot was used to detect cytoplasmic and nuclear protein of p53, total protein of AMP-activated protein kinase (AMPK), mammalian target of rapamycin (mTOR) and Light chain 3 (LC3). Quantitative Real-Time PCR (qRT-PCR) was used to detect p53 mRNA level after treated with IL-7. Then using transmission electron microscopy to observe the morphological change of autophagosome. 123 cases of NSCLC were collected for survival analysis, immunohistochemistry staining and cox regression multivariate analysis. We find that IL-7 induce the p53 translocation from nucleus to cytoplasm, then IL-7 down-regulate phosphorylation of AMPK and up-regulate phosphorylation of mTOR. The expression of AMPK and p53 were associated with IL-7/IL-7R and mTOR expression. Clinically, AMPK and p53 were well correlated with stage and survival of lung cancer patients. IL-7R, mTOR and tumor stage were the strongest predictors of survival. In conclusion, IL-7 inhibit autophagy in NSCLC via P53 regulated AMPK/mTOR signaling pathway. AMPK and p53 are correlated with patients’ survival. IL-7R, mTOR and tumor stage are the strongest predictor of survival.

## Introduction

Over the years, Lung cancer still be the most threatening cancer in the world, for low survival rate (19%) and lack of effective screening method^1^. Lung cancer are mainly classified into two types: non-small cell lung cancer (NSCLC) and small cell lung cancer (SCLC). Interleukin 7 (IL-7), a member of IL-2 super-family, is found to be essential in the development of B cell and T cell. Oddly, IL-7 seems play dual role in tumors. It is reported that IL-7 took anti-tumor effect in prostate cancer, glioma, melanoma, leukemia, and lymphoma. While IL-7 present pro-proliferation, promote lymphangiogenesis, and anti-apotosis in NSCLC; increasing invasion and migration in bladder cancer cell; besides, breast cancer with higher grade and malignancy are associated with higher expression of IL-7^2–5^. Thus, the role of IL-7 in cancer are still elusive.

Autophagy (mainly represent for macroautophagy) is a self-eating process that transport the organelles and protein to autophagosome and fuses with lysosome. These protein can be degraded and then reused by cell for metabolism^6^. It is reported that cancer cell frequently observed with high level of autophagy which may essential for their proliferation and cope with stress^7^.

There are many autophagy related gene (ATG) and important signaling markers in autophagy regulation. In our study, we will find out whether IL-7 regulate autophagy via p53, AMP-activated protein kinase (AMPK), mammalian target of rapamycin (mTOR) and light chain 3 (LC3, also known as mammalian homolog of Atg8)^8^ in NSCLC cell line.

Despite enormous study discuss about p53 play a role in autophagy. However the relationship between different status of p53 and autophagy are still elusive. It is reported that P53 activates autophagy and autophagy suppress p53, they are functionally intertwined^9^. Many previous research showed that the localization of p53 appear to play dual role in autophagy. Cytoplasmic P53 inhibit autophagy, while nuclear p53 promote autophagy^6,10^. AMPK, a serine/threonine protein kinase, is composed of α,β,γ subunits and regulating energy status, such as glucose or lipid metabolism and mitochondrial biogenesis^11^. Besides, AMPK promote autophagy via antagonize mTORC1 complexes and activate ULK1 complexes^11,12^. But which is the upstream factor between p53 and AMPK is not known. In our study, A549 (p53 wild type) cell line was employed to unveil whether IL-7 regulate autophagy via p53, AMPK and mTOR. In addition, we will explore whether AMPK, P53 expression correlated with IL-7/IL-7R level, clinical stages, and NSCLC patient survival.

## Material and methods

### Cell culture and in vitro treatment

All experiments were performed in accordance with relevant guidelines and regulations. No live animals were used in this study. A549 cell line was purchased from Cell Bank of the China Academy of Sciences (Shanghai, China) and maintained in RPMI 1640 (Gibco, Waltham, MA, USA). Media were supplemented with 10% fetal bovine serum (ExCell Bio,Shanghai, China, FSP500) and 100 U/ml penicillin (Sigma-Aldrich, St. Louis, MO, USA), 100 μg/ml streptomycin (Sigma-Aldrich, St. Louis, MO, USA). Cell were cultured at 37 °C in a humidified atmosphere containing 5% CO2 until 80% confluent. Firstly, A549 cells incubated with IL-7 (24 ng/ml) for different times at 0 h, 8 h and 12 h. Then A549 cell line were divided into several groups: control group; IL-7 (24 ng/ml) stimulating 12 h (12 h) group; anti-IL-7 receptor (A-IL-7R) group (cells incubated with A-IL-7R monoclonal antibody 100 ng/ml plus IL-7 24 ng/ml); p53 suppressing group: cells were incubated with pifithrin‐α (PFT-α)(30 μmol/ml) for 12 h; PFT-α and IL-7 group: cell preincubated with PFT-α for 2 h then incubated with IL-7; AMPK inhibiting group: cell were incubated with Compound C (dorsomorphin)(30 μmol/ml) for 12 h; and Compound C plus IL-7: cells pretreated with Compound C for 2 h then treated with IL-7; mTOR suppressing group: cells were treated with Rapamycin (100 ng/ml) for 12 h, Rapamycin plus IL-7 group: cells pretreated with rapamycin for 2 h then treated with IL-7.

### Reagents and antibody

IL-7 was purchased from Peprotech (Peprotech, Rocky Hill, NJ). P53 (1:1000, Proteintech Group, Chicago, IL, USA), AMPK-α (1:1000, Wanlei Bio, Shenyang, China), β-actin (1:1000, Proteintech Group,Chicago, IL, USA), GAPDH (1:1000, Proteintech Group,Chicago,IL, USA), anti-mouse IgG (1:2000, Proteintech Group, Chicago, IL, USA) and anti-rabbit IgG (1:2000, Proteintech Group, Chicago, IL, USA), mTOR (1:1000, Cell Signaling Technology, Beverly, MA), phosphorylated(p)-mTOR (1:1000, Cell Signaling Technology, Beverly, MA) and p-AMPK (1:1000, Cell Signaling Technology, Beverly, MA), LC3B (1:1000,Cell Signaling Technology, Beverly, MA), anti-IL-7 receptor antibody (A-IL-7R) (Santa Cruz, CA), P53 inhibitor: PFT-α (MedChemExpress, Monmouth, NJ, USA), AMPK inhibitor: Compound C (MedChemExpress, Monmouth, NJ, USA) and mTOR inhibitor: Rapamycin (MedChemExpress, Monmouth, NJ, USA).

### Western blot analysis

After IL-7 stimulation, total protein were extracted with NP-40 lysis buffer (Beyotime Biotechnology, Shanghai, China) (containing 1× protease and phosphatase inhibitors cocktail) on ice for 25 min. Then centrifuged at 12,000 rpm for 20 min at 4 ◦C. After quantified, total protein sample (40 μg protein per lane) were electrophoresed on 8% and 12% SDS polyacrylamide electrophoresis (SDS-PAGE) gel. Before transferred to polyvinylidene difluoride (PVDF) membrane (Millipore, Billerica, MA, USA), gel were cropped for each specific antibody according to their protein molecular weight. Then, membrane were incubated with specific primary antibody in 4 °C overnight and secondary antibody for 2 h at room temperature. Finally, membrane were visualized using electrochemiluminescence (ECL) (Wanlei, Shenyang, China) and detected with BioImaging System (UVP Inc., Upland, CA, USA).

Cytoplasmic and nuclear protein were extracted by using cytoplasmic and nuclear protein extraction kit (Beyotime Biotechnology, Shanghai, China) according to manufacturer’ s protocol. Nuclear protein (30 μg) and cytoplasmic protein (30 μg) were electrophoresed on 8% SDS-PAGE gel then transferred to PVDF membrane. Other process are similar as previously described.

### RT-qPCR

Sample were isolated by trizol, then 1 μg RNA were reverse transcribed into single-strand cDNA using PrimeScript RT reagent kit (Takara, Tokyo, Japan, RR047A). Finally SYBR Green Real Time PCR master mix (Takara, Tokyo, Japan, RR820A) was used to perform in 7900HT Fast Real‐Time PCR System (Applied Biosystems, Foster City, CA, USA). Parameter of PCR reaction are as follows: incubation at 95 °C for 30 s, followed by 40 cycles of 95 °C for 5 s, 60 °C for 34 s, finally 95 °C for 15 min, 60 °C for 1 min and 95 °C 15 s. Gene expression level were calculated by the 2^-ΔΔCT^ method. Primer sequences are as follows:p53 forward: 5′-GCTGAGTATCTGGACGACAGG-3′; p53 reverse: 5′-AGCGTGATGATGGTAAGGATG-3′, β‐actin forward: 5′‐ATAGCACAGCCTGGATAGCAACGTAC‐3′; β‐actin reverse: 5′‐CACCTTCTACAATGAGCTGCGTGTG ‐3′.

### Transmission electron microscopy

After A549 cell were treated corresponding to the experimental design described above, TEM was used to observe autophagosome in A549 cell. After cell were washed with phosphate buffer saline (PBS), they were digested with 0.25% trypsin and centrifugation in 1.5 ml tube. Then sediment were fixed with glutaraldehyde for 2 h in 4 °C. Subsequently, sample were post-fixed with osmium and dehydrated with ethanol. Finally, sample examined via TEM (Hitachi, Tokyo, Japan).

### Patients and specimens

A total of 123 cases of NSCLC were obtained from the 1st January 2010 to the 31st December 2020 at General Hospital of Northern Theater Command, Shenyang, China. Study was approved by General Hospital of Northern Theater Command Ethics Committee. The tumour tissues in this study were from patients who had NSCLC proved by pathological diagnosis without distant metastasis. None of the 123 cases had received radiation therapy or chemotherapy before surgery. The TNM staging system of the UICC (2017) was used to classify the specimens.

The survival time was calculated from the operation day to death via the evaluation of recurrence and metastasis or until the last follow-up date (December 2013). The following-up of the surviving patients averaged 27.09 months and ranged from 1 to 156 months. The study has been approved by the General Hospital of Northern Theater Command Ethics Committee.

### Immunohistochemistry

Four-micron thick sections were prepared from the paraffin-embedded tissues. Immunostaining was performed by the streptavidin-peroxidase (S-P) method (Zhongshan, Beijing, China). The primary antibodies were anti-IL-7, anti-IL-7R, anti-P53, anti-AMPK and anti-mTOR (1:100, 1:100, 1:150, 1:100, 1:150) antibodies. The peroxidase reaction was developed with DAB. For negative control, the primary antibodies were replaced by non-immune serum.

All the samples were evaluated by two independent pathologists. The staining intensity and extent of IL-7, IL-7R, P53, AMPK, and mTOR were scored as follows. Staining intensity was defined as the depth of shade of the protein and graded as 0 (no staining), 1 (mild staining), 2 (moderate staining), 3 (strong staining). Staining extent was defined as the percentage of tumor cells with positive brown-yellow staining and graded as follows: 0 (< 10%), 1 (10–49%), 2 (≥ 50%). Then sum index of the two variables was obtained and a total score of 4 was considered as the point to categorize the staining as high (≥ 4) or low (< 4)^11–13^.

### Statistical analysis

SPSS19.0 (SPSS incorporated, Chicago) was used for all analysis. One-way analysis of variance (ANOVA) was performed for statistical analysis, and post hoc analysis Dunnett's test was used for each group compared to control. *P*-value < 0.05 was considered statistically significant. All the experiments were conducted in triplicate. The Chi-square test was used to analyze the relationship between P53 and AMPK expression and clinical pathological factors; these tests were also used to evaluate whether there was any significant relationship between P53/AMPK and IL-7/IL-7R; Kaplan–Meier curves were used for survival analysis, and log-rank was determined based on the differences; Cox regression multivariate analysis was used to evaluate P53 and AMPK as prognostic factors to compare them with other strong prognostic factors for prognosis in lung cancer.

### Ethics approval

The study has been approved by the General Hospital of Northern Theater Command Ethics Committee.

### Approval for human experiments

1. Our study was approved by General Hospital of Northern Theater Command Ethics Committee. 2. Confirms that all experiments were performed in accordance with relevant named guidelines and regulations. 3. Written informed consent was exempted by Northern Theater Command Ethics Committee because we used retrospective sample and data, and our study obey the ethics regulation.

## Results

### IL-7 induced the p53 translocation from nucleus to cytoplasm

**Figure 1:**
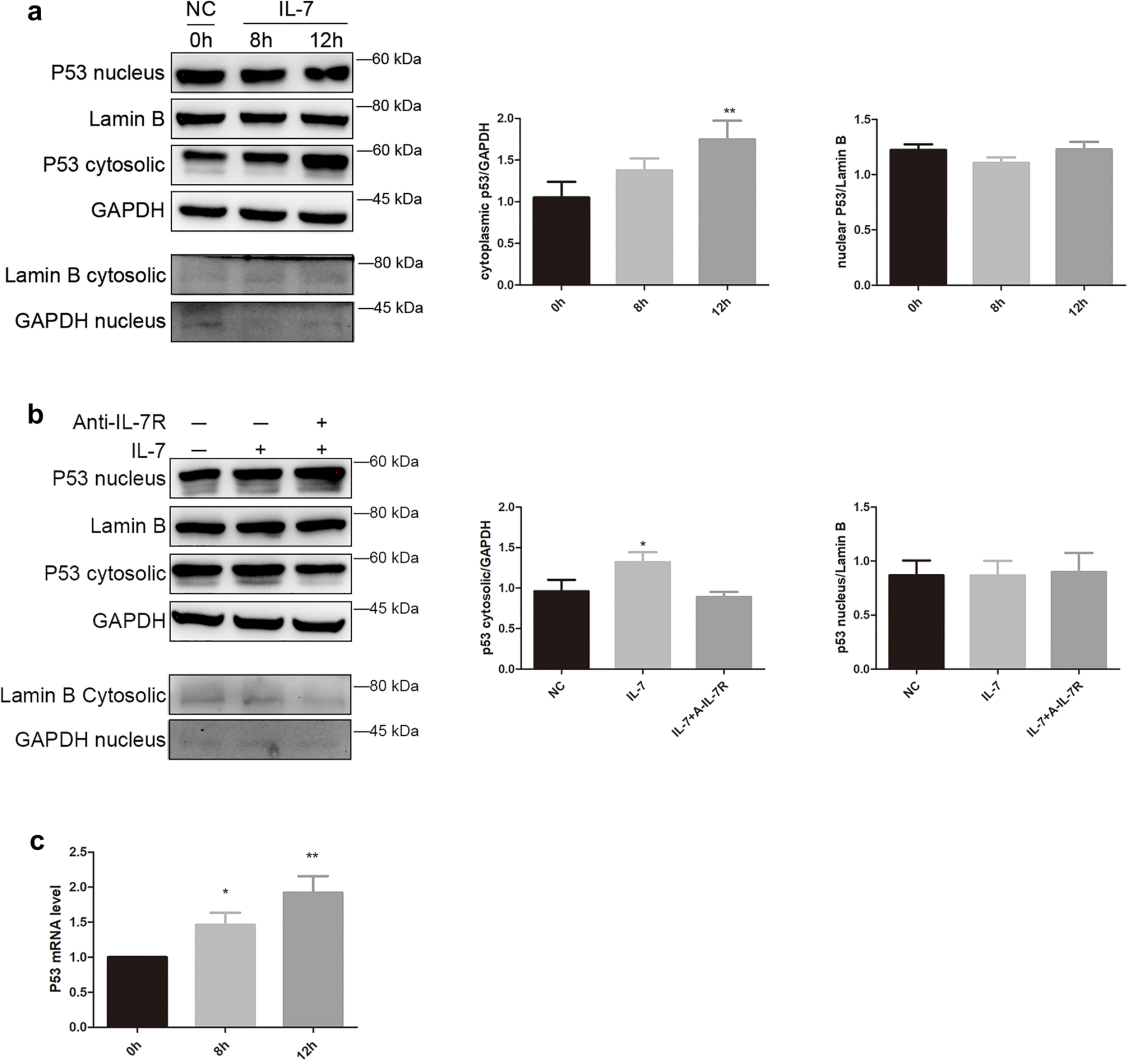
Isolation of cytoplasmic and nuclear p53 protein for A549 cells after treated with interleukin 7 (IL-7), anti-IL-7 receptor antibody (A-IL-7R) for western blot analysis. Then total RNA was isolated for real-time quantitative PCR (RT-qPCR) assay after incubated with IL-7 for different time. (**a**) A549 cells were treated with IL-7 at 24 ng/ml for 8 h and 12 h, then p53 nuclear and cytoplasmic protein were detected. Lamin B was used as nuclear loading control and GAPDH was used for cytoplasmic loading control. Cytoplasmic Lamin B and nuclear GAPDH were detected to validate nuclear protein was not contaminated by cytoplasmic protein and cytoplasmic protein were not contaminated by nuclear protein. Bar show the cytoplasmic and nuclear p53 western blot analysis. (**b**) A549 cells were treated with IL-7 and A-IL-7R plus IL-7 for 12 h, then nuclear and cytoplasmic p53 protein were detected by western blot. Cytosolic Lamin B and nucleus GAPDH was detected as negative control. Bar show the cytoplasmic and nuclear p53 western blot analysis. (**c**) After incubated with IL-7 at different time (0 h, 8 h, 12 h), p53 mRNA level was detected by RT-qPCR, all the bar graph expressed as Mean ± SD, n = 3. *represent for *P* < 0.05 versus negative control (NC). **represent for *P* < 0.01 versus NC.

We isolated nuclear and cytoplasmic protein of A549 at 8 h, 12 h and 24 h (Fig. 1a). IL-7 exert its peak effect at 12 h, thus we chose 12 h as optimal incubation time in our sequential experiments. After IL-7 treated for 12 h, we observed a notably increase of cytoplasmic p53 (Fig. 1a), so as an escalation of p53 mRNA level (Fig. 1c). This effect could be blocked by anti-IL-7 receptor antibody (A-IL-7R) (Fig. 1b). Cytosolic Lamin B and nucleus GAPDH protein expression almost negative illustrate cytoplasmic and nuclear protein extraction kit is viable and our application method is correct (Fig. 1a,b). These data suggest IL-7 affected p53 distribution between nuclear and cytoplasm in A549 cell line.

### IL-7 affect phosphorylated form of AMPK and mTOR

**Figure 2:**
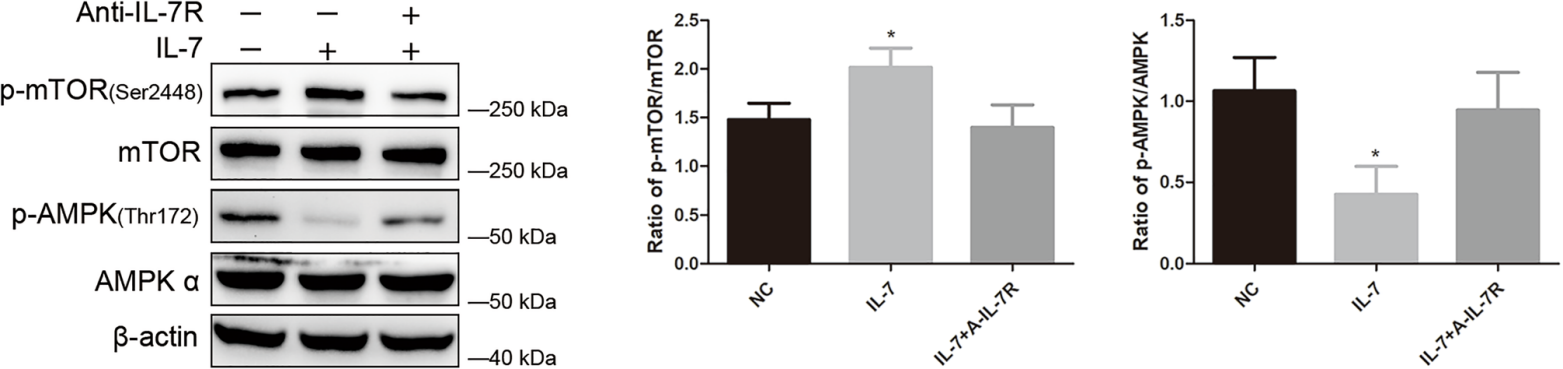
IL-7 regulating AMPK/mTOR pathway. Western blot analysis show down-regulation of phosphorylated (p)-AMPK proteins and up-regulation of p-mTOR protein in A549 cells after treated with IL-7, and using A-IL-7R specific antibody block the effect of IL-7, however the expression level of total AMPK and mTOR are not significantly affected. All the bar graph expressed as Mean ± SD, n = 3. *represent for *P* < 0.05 versus negative control (NC). **represent for *P* < 0.01 versus NC.

We detected total protein of A549 cells after incubated with IL-7 and IL-7 plus its A-IL-7R antibody: the result show that p-AMPK decreased and p-mTOR increased, while total protein of AMPK and mTOR did not change, and this effect could be blocked by A-IL-7R, which indicate that IL-7 affect p-AMPK and p-mTOR (Fig. 2).

### IL-7 regulate mTOR via AMPK

**Figure 3:**
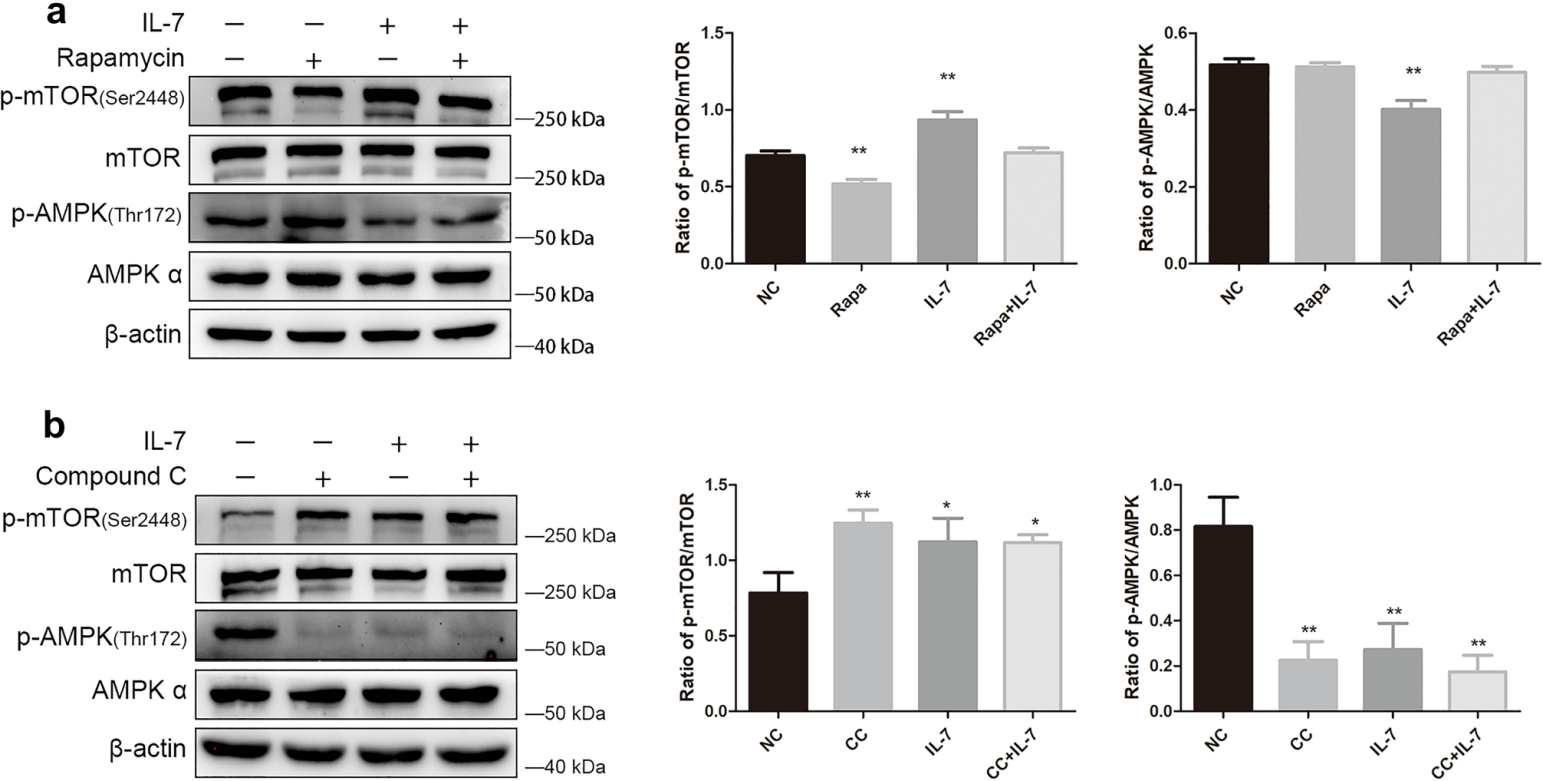
IL-7 regulate mTOR via AMPK. (**a**) Rapamycin manipulate the down-regulation of p-mTOR/mTOR but had no effect on p-AMPK/AMPK. Besides, Rapamycin impede IL-7 regulated p-mTOR up-regulation. (**b**) Compound C down-regulate the p-AMPK/AMPK expression meanwhile escalate p-mTOR/mTOR expression. IL-7 and Compound C had similar effect on A549 cell. The bar graph corresponding to their western blot results show different kind of protein analysis after cell treated with IL-7, Rapamycin IL-7 + Rapamycin, Cpmpound C and IL-7 + Compound C, then compared with negative control. Mean ± SD, n = 3. *represent for *P* < 0.05. ** and *** represent for *P* < 0.01 versus NC.

We use Compound C (AMPK inhibitor) and Rapamycin (mTOR inhibitor) to investigate the relationship between AMPK and mTOR. After treated with Rapamycin, in Western Blot analysis, seldom do we observe the change of p-AMPK protein (Fig. 3a), but we observed a dramatically p-mTOR suppression (Fig. 3a), which indicate mTOR is not the up-regulator for AMPK. Then, cells were treated with Compound C, results show that Compound C shut down the p-AMPK/AMPK expression and escalate the p-mTOR/mTOR expression level (Fig. 3b). All these results indicate that AMPK is the upstream factor for mTOR and IL-7 regulate mTOR via AMPK.

### IL-7 regulate AMPK and mTOR via p53

**Figure 4:**
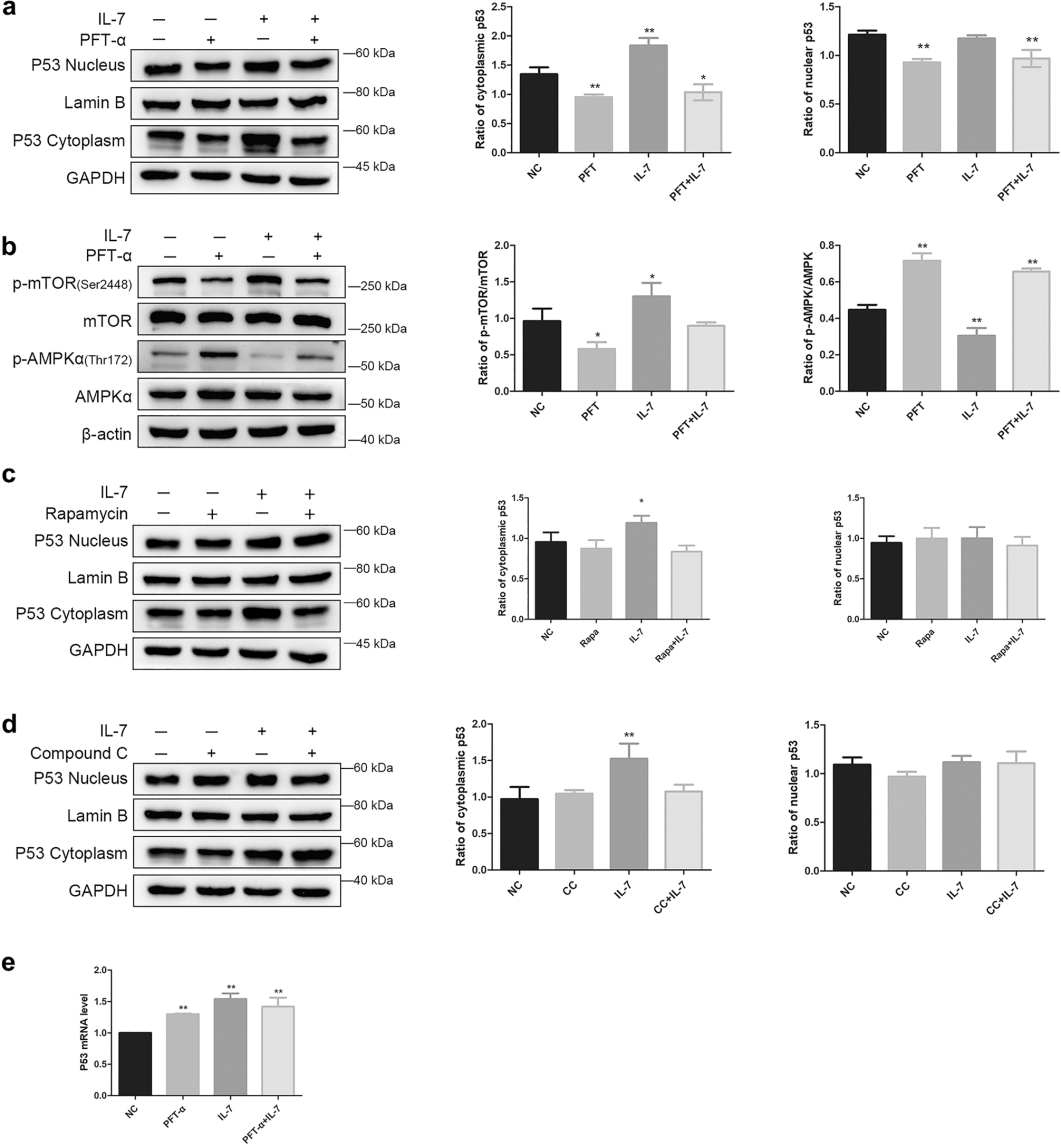
IL-7 regulate AMPK and mTOR via P53. (**a**) Cells were treated with PFT-α, western blot analysis show nucleus and cytoplasmic p53 slightly decrease, besides, PFT-α blunt the effect of IL-7 modulated p53 translocation from nucleus to cytoplasm. (**b**) PFT-α cause the up-regulation of p-AMPK/AMPK and down-regulation of p-mTOR/mTOR, and impede the effect of IL-7 on A549 cell. (**c**) Cell treated with Rapamycin, neither nuclear p53 nor cytoplasmic p53 were affected. (**d**) Cell incubated with Compound C, there is no significant change on p53 nucleus and cytoplasm. (**e**) qRT-PCR show both PFT-α and IL-7 cause increase of p53 mRNA level. All the bar graph expressed as Mean ± SD, n = 3. *represent for *P* < 0.05 versus negative control (NC). **represent for *P* < 0.01 versus NC.

Firstly, We added PFT-α (p53 inhibitor) to further explore relationship between p53, AMPK and mTOR. As shown in (Fig. 4a,e), IL-7 manipulate an increase of cytoplasmic p53 protein and p53 mRNA level, however there is no obvious change in p53 nuclear protein and these effect can be blunt by PFT-α (Fig. 4a). Meanwhile, PFT-α cause a significant up-regulation of p-AMPK and down-regulation of p-mTOR (Fig. 4b). Then, rapamycin was used to observe the change of cytoplasmic and nuclear p53 protein. As shown in Fig. 4c, seldom do we observe change neither nuclear p53 protein nor cytoplasmic p53 protein, which indicate that mTOR is not the upstream regulator for p53. Last bu not least, Compound C was used, western blot results show that Compound C can not regulate p53 protein neither nucleus nor cytoplasm (Fig. 4d), which demonstrate AMPK is not the up-stream factor of p53. All these results indicate that IL-7 regulate AMPK and mTOR via p53.

### IL-7 inhibit autophagy of A549

**Figure 5:**
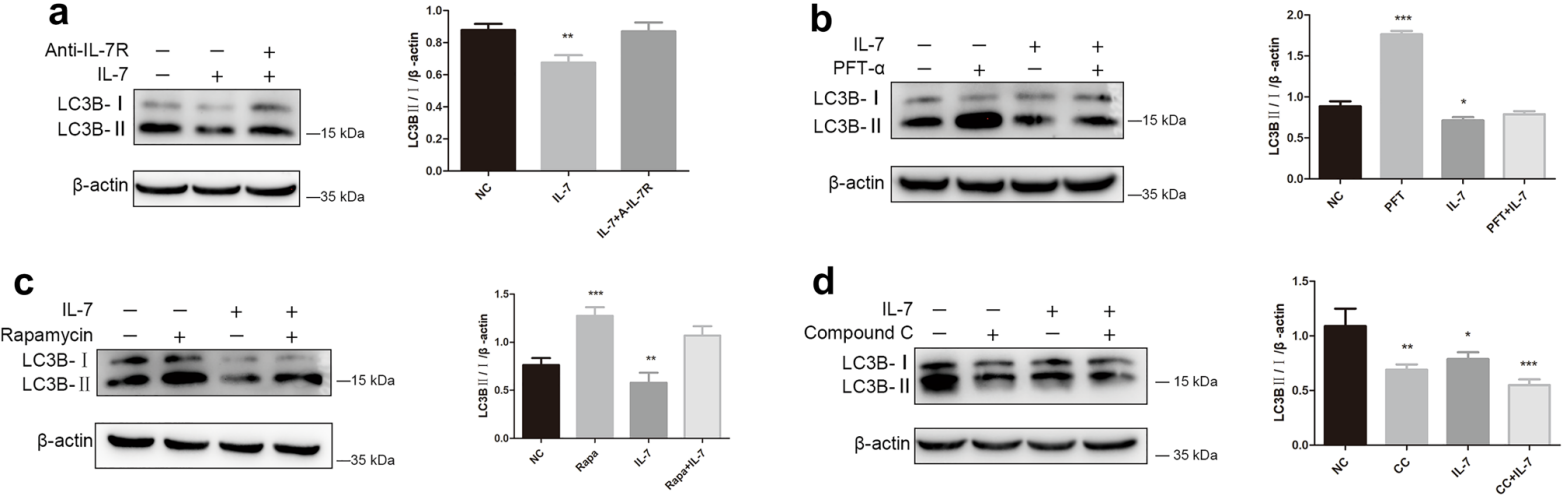
IL-7, PFT-α, Rapamycin, Compound C affect LC3 expression. (**a**) IL-7 down-regulate LC3 and this effect can be blunt by IL-7R. (**b**) PFT-α up-regulate the LC3 protein level. (**c**) Rapamycin increase the expression of LC3. (**d**) Compound C decrease the expression of LC3. All the bar graph expressed as Mean ± SD, n = 3. *represent for *P* < 0.05 versus negative control (NC). ** and *** represent for *P* < 0.01 versus NC.

**Figure 6:**
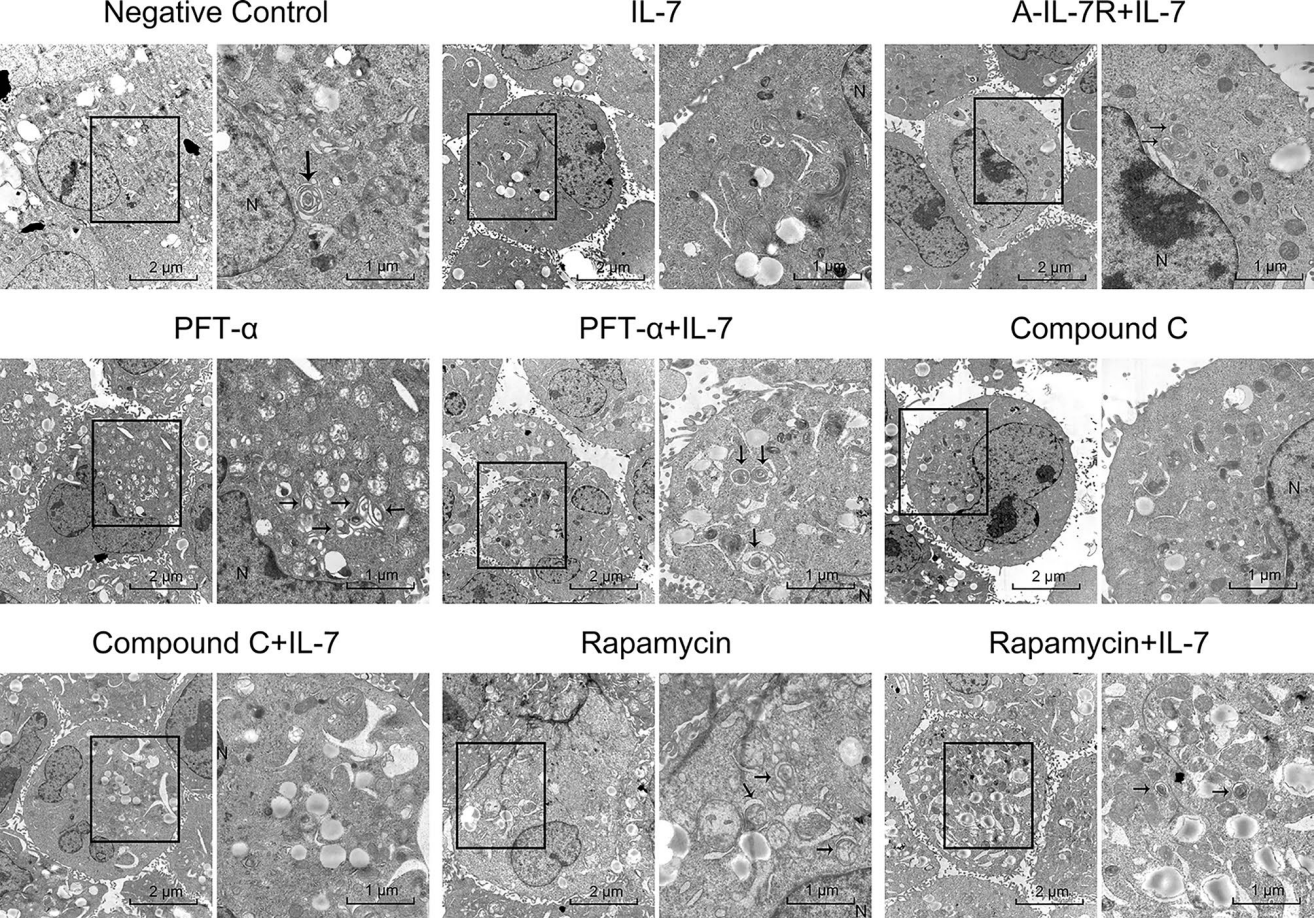
Transmission Electron Microscopy (TEM) results show the group as designed above. Images were taken at either 8000× or 20,000×. The 20,000× image was the enlarged image of 8000× in the black frame. In the 20,000×, the black arrow indicate characteristic autophagosome in A549 cells after treated with IL-7 (autophagosome decrease); cells treated with pifithrin‐α (autophagosome notably increase); cells treated with pifithrin‐α + IL-7 (autophagosome increase); cells treated with Compound C (autophagosome decrease); cells incubated with Compound C + IL-7 (autophagosome decrease); cells treated with A-IL-7R antibody + IL-7 (effect of IL-7-induced autophagosome depression was blunt); cells incubated with Rapamycin (autophagosome increase) and cells treated with IL-7 + Rapamycin (autophagyosome slightly increase).

To confirm that IL-7 regulate autophagy suppression, western blot were used to analyze LC3-II/I protein level andTEM were applied to observe autophagosome as the group previously designed: negative control group, cells treated with IL-7, IL-7 + A-IL-7R, Rapamycin, Rapamycin + IL-7 Compound C, Compound C + IL-7, PFT-α, and IL-7 + PFT-α. Western blot for LC3 show that IL-7 decrease LC3II/I protein level and this can be blocked by A-IL-7R (Fig. 5a). In addition, PFT-α and Rapamycin increase the LC3II/I (Fig. 5b,c) and Compound C decrease the LC3II/I level (Fig. 5d). Similar results were obtained in TEM assay: IL-7 group, Compound C group and IL-7 + Compound C group find out a decrease of autophagosome; Rapamycin group, Rapamycin + IL-7 group, PFT-α and IL-7 + PFT-α group observed a noteworthy increase of autophagosome; control group observed an basic autophagosome level in A549 cells and the autophagosome level of IL-7 + A-IL-7R group is similar to control group (Fig. 6). Both the LC3 and TEM results indicate IL-7 inhibit autophagy of A549 cell line.

### Expression of AMPK and P53 correlate with IL-7/IL-7R level, clinical stages, and NSCLC patient survival

**Figure 7:**
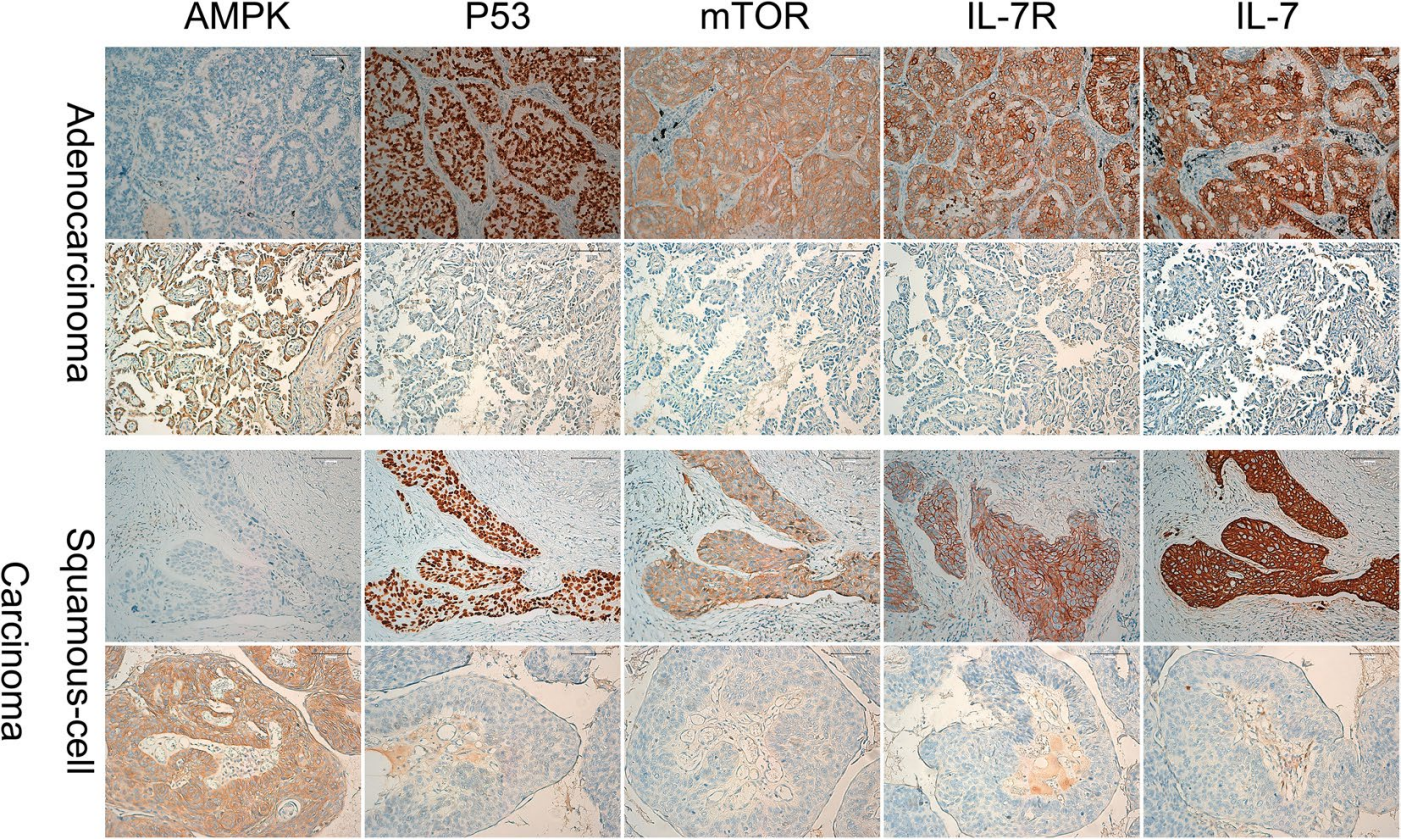
In both adenocarcinoma and squamous-cell carcinoma, AMPK low expression correlates with high expression of P53, mTOR, IL-7 and IL-7R. AMPK high expression associated with low expression of P53, mTOR, IL-7 and IL-7R. Meanwhile, The high expression of P53 was significantly associated with the high expression of IL-7, IL-7R, mTOR, and AMPK low expression.

**Figure 8:**
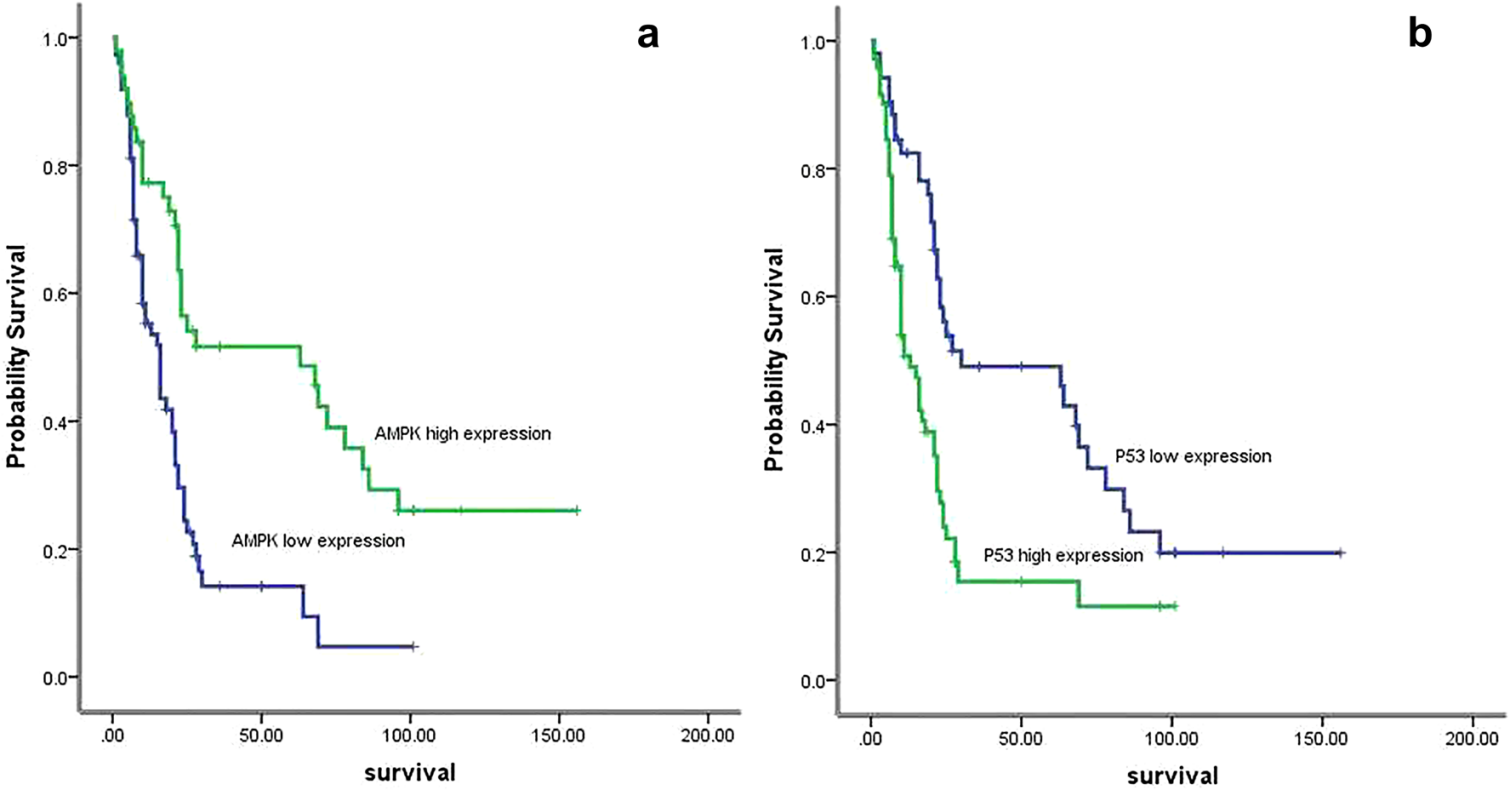
Expression of AMPK and p53 correlates with survival of NSCLC patients. (**a**) AMPK high expression had a longer survival than those with AMPK low expression. (**b**) P53 low expression had a longer survival compared with P53 high expression.

Immunohistochemical analysis of 123 NSCLC specimens revealed that the frequency of high expression of AMPK was 39.84% (49/123). The high expression of AMPK was significantly associated with the low expression of IL-7, IL-7R, mTOR, and P53, on the contrary, low expression of AMPK are associated with high expression of IL-7, IL-7R, mTOR, and P53 (Table 1, Fig. 7). Clinically, compared to the NSCLC with high expression of AMPK, tumors with low expressions of AMPK were more advanced-stage cancer (*P* < 0.001). Patients with high expression of AMPK (median survival = 63 ± 24.92 months; 95% confidence interval 14.157–111.843 months) had a statistically significant longer survival than those with low expression of AMPK (median survival = 16 ± 2.608 months; 95% confidence interval 10.888–21.112 months; *P* < 0.001; Fig. 8a).

**Table 1:** Relationship between AMPK expression in NSCLC and clinical pathological factors (Chi-square test).

	Patients	AMPK high expression (%)	*P* value	χ^2^ value
**Gender**				
Male	89	35 (39.33%)		
Female	34	14 (41.18%)	1.000	0.035
**Age**				
≤ 60	71	30 (42.25%)		
> 60	52	19 (36.54%)	0.579	0.409
**Histology**				
Squamous cancer	72	27 (37.5%)		
Adenocarcinoma	51	22 (43.14%)	0.578	0.396
**Differentiation**				
Well	16	6 (37.5%)		
Moderate	55	24 (43.64%)		
Poor	52	19 (36.54%)	0.739	0.604
**Stage**				
I–II	56	32 (57.14%)		
III	67	17 (25.37%)	0.000	12.846
**mTOR expression**				
High	72	16 (22.22%)		
Low	51	33 (64.71%)	0.000	22.481
**P53 expression**				
High	71	19 (26.76%)		
Low	52	30 (57.69%)	0.001	11.983
**IL-7 expression**				
High	89	24 (26.97%)		
Low	34	25 (73.535%)	0.000	22.255
**IL-7R expression**				
High	81	28 (34.58%)		
Low	42	21 (50%)	0.000	19.154

However, the frequency of high expression of P53 was 57.72% (71/123). The high expression of P53 was significantly associated with the high expression of IL-7, IL-7R, mTOR, and AMPK low expression (Table 2, Fig. 7). The high expression level of P53 was significantly associated with clinical stage of tumors (*P* = 0.01). In addition, patients with low expression of P53 (median survival = 30 ± 22.5 months; 95% confidence interval 0–74.1 months) survived longer than those with high expression of P53 (median survival = 13 ± 2.174 months; 95% confidence interval 8.74–17.26 months; *P* < 0.001; Fig. 8b).

**Table 2:** Relationship between P53 expression in NSCLC and clinical pathological factors (Chi-square test).

	Patients	P53 high expression (%)	*P* value	χ^2^ value
**Gender**				
Male	89	47 (52.81%)		
Female	34	24 (70.59%)	0.102	3.187
**Age**				
≤ 60	71	41 (57.75%)		
> 60	52	30 (57.69%)	1.000	0.000
**Histology**				
Squamous cancer	72	41 (56.94%)		
Adenocarcinoma	51	30 (58.82%)	0.855	0.043
**Differentiation**				
Well	16	9 (56.25%)		
Moderate	55	33 (60%)		
Poor	52	29 (55.77%)	0.899	0.212
**Stage**				
I–II	56	25 (44.64%)		
III	67	46 (68.66%)	0.01	7.208
**mTOR expression**				
High	72	51 (70.83%)		
Low	51	20 (39.22%)	0.001	12.229
**AMPK expression**				
High	49	19 (38.78%)		
Low	74	52 (70.27%)	0.001	11.983
**IL-7 expression**				
High	89	62 (69.66%)		
Low	34	9 (26.47%)	0.000	18.807
**IL-7R expression**				
High	81	58 (71.6%)		
Low	42	13 (30.95%)	0.000	18.731

The expression levels of P53 and AMPK protein were not associated with a patient's age or sex, the tumor histological type, and differentiation (Tables 1, 2). In the Cox regression multivariate analysis, IL-7R, mTOR, and tumour stage were the strongest predictors of survival (Table 3).

**Table 3:** Multivariate Cox regression mode.

	Wald	Sig	Exp (B)	95% CI for Exp (B)
Sex	0.066	0.798	0.932	0.546–1.591
Age	0.136	0.713	0.919	0.587–1.439
Histology	0.109	0.741	1.084	0.672–1.748
Differen	1.918	0.383		
Differen (1)	0.262	0.609	0.826	0.398–1.716
Differen (2)	1.917	0.166	0.705	0.431–1.156
Stage	20.685	0.000*	0.296	0.175–0.500
IL-7	0.521	0.47	0.711	0.281–1.796
IL-7R	14.582	0.000*	0.160	0.062–0.409
mTOR	4.076	0.043*	0.539	0.296–0.982
P53	0.611	0.434	1.236	0.726–2.104
AMPK	0.054	0.816	0.932	0.516–1.684

## Discussion

Autophagy plays dual role in cellular activities. On the one hand, by degrading and reusing damaged cellular organelles, autophagy maintain cellular survival and metabolism^6^. Some cancer cell lines, such as pancreatic cancer cells, have abnormally high autophagy level^6^. It is likely that autophagy is required for cancer aggression^14^. On the other hand, BECN1 (Beclin-1), an autophagy associated gene which play a role in autophagosome formation, are frequently deleted in some cancer, such as breast cancer, ovarian and prostate cancer^15^. Other autophagy proteins are observed aberration and expression alteration. There is an emerging hypothesis that autophagy may act as anticarcinogenesis in the beginning status of oncocytoma^15^, but once tumor is established, autophagy may increase for cancer cell survival and fight against stress^15,16^.

The role of IL-7 in tumor apoptosis, migration, proliferation and lymphangiogenesis has been reported. But its role in autophagy are still elusive. In a recent study, Jifeng Zhu etc. revealed that IL-7 inhibit macroautophagy in schistosome infected mice and this effect of IL-7 may exacerbated liver pathology^17^. In our latest study, we find IL-7 suppress formation of autophagosome in NSCLC, mainly by regulating AMPK/mTOR signaling pathway via translocation of nuclear/cytoplasmic p53 and regulating PI3K/AKT/mTOR signaling pathway via Beclin-1^18^. In addition, the effect of IL-7 could be blocked by A-IL-7R antibody.

Autophagy or glucose restriction leads to p53 degradation or suppression, but this effect are associated with p53 status^9,19^. Interestingly, there is also evidence suggest that p53 activate autophagy, for p53 directly or indirectly targeting autophagy related gene such as DRAM, ULK1 and Atg7^9^. Cytoplasmic p53 inhibit augophagy while nuclear p53 promote autophagy^6^. But the mechanism of how does different status of p53 affect autophagy are not known. To further interrogate the effect of different p53 status on the autophagy modulation, A549 cell line (p53 wild type) were chose. Previously, we reported that IL-7 regulate autophagy by PI3K/AKT/mTOR signaling pathway via Beclin-1^18^ and prevent apoptosis via p53^20^. IL-7 caused up-regulation of p-mTOR and change of p53. Thus, we asked whether p53 get involved in IL-7 regulated mTOR pathway and autophagy. By isolation of cytoplasmic and nuclear protein of p53 we find IL-7 cause p53 translocation from nucleus to cytoplasm at 12 h. we also observed an increase of p53 mRNA level by using RT-qPCR. Despite seldom do we observed suppression in p53 nuclear protein, the p53 mRNA level and p53 cytoplasmic protein do escalated. We speculate that p53 protein mainly express in nucleus in NSCLC, just a small part locate in cytoplasm. Thus a slightly shut down of p53 protein in nucleus were not detected by western blot explicitly, but a mildly increase in cytoplasm were observed. An increase of p53 mRNA validate IL-7 induce p53 expression. Then, by detecting total protein of AMPK and mTOR and its phosphorylated form, we observed IL-7 caused decrease of p-AMPK and increase of p-mTOR.

Mechanistically, several molecular, such as AMPK, DRAM, DAPK-1, PTEN, IGF-BP3 have been reported as downstream factor of p53^21^. It is reported that some kinases, including CAMKK, LKB1, TGF-β-activated protein kinase 1 (TAK1), and ataxia telangiectasia mutated (ATM) kinase play a role as upstream factor of AMPK^22^. However, whether p53 regulate AMPK or AMPK affect p53 are still elusive^23^. mTOR is a core component of several distinct complexes including mTOR complexes 1 (mTORC1), mTOR complexes 2 (mTORC2), and a putative mTOR complexes 3 (mTORC3). These complexes interact with several kinase and protein such as AMPK, PI3K-Akt, serum and glucocorticoid-induced kinase (SGK)^24^. mTOR participated in many cellular behaviors, such as autophagy, apoptosis, transcription, as well as cell growth, cell proliferation, survival^25^. Our experiment conducted a serial of inhibitor to substantiate the relationship between p53, AMPK and mTOR. After incubated with PFT-α (p53 inhibitor), western blot results show that both nuclear and cytoplasmic p53 protein decreased, then we observed increase of p-AMPK expression level and decrease of p-mTOR level. Meanwhile, PFT-α block the effect of IL-7-regulated autophagy suppression. These results elucidate IL-7 regulate p53 translocate from nucleus to cytoplasm and p53 affect AMPK and mTOR. Compound C (AMPK inhibitor) could up-regulate p-mTOR expression and inhibit autophagy, but p53 protein level was not affected, which indicate AMPK is downstream factor of p53. Rapamycin (mTOR inhibitor) cause p-mTOR attenuation and autophagy activation, but had no effect on p53 and AMPK, illustrating p53 and AMPK regulate mTOR. These results demonstrated IL-7 inhibit autophagy via p53 regulated AMPK/mTOR signaling pathway in A549 cell line.

LC3, the mammalian homologue of yeast Atg8, is localized in autophagosome membrane and commonly considered as autophagy marker^25,26^. But sometimes an increase in autophagosome does not correlate with LC3 aggregate^27^. Thus TEM is another reliable way to assess autophagy level. In our work, both LC3II/I protein level and TEM results indicate that IL-7 inhibit autophagy in A549 cell, and these effect can be blocked by A-IL-7R. In addition, PFT-α notably activate autophagy demonstrating that p53 suppressing lead to autophagy activation. In line with this, mTOR inhibition also cause autophagy induction. Besides, AMPK inhibiting cause autophagy suppression.

In our study, immunohistochemical analysis of 123 NSCLC specimens show that AMPK low expression are associated with p53, mTOR, IL-7, IL-7R high expression. Clinically, patients with AMPK low expression are more associated with advanced-stage cancer (*P* < 0.001) and patients with high expression of AMPK had a statistically significant longer survival than those with low expression of AMPK. Similar to our findings, Nan Li et al. find out in both squamous cell carcinoma and lung adenocarcinoma, p-AMPK low expression had a higher survival rate^28^. However, Daohui Gong et al. demonstrated that AMPKα1 was highly expressed in NSCLC cancer tissues and correlated with poor prognosis in patients with NSCLC^29^. The immunohistochemical analysis of p53 showed the opposite results with AMPK. Zhihua Teng et al. believe that lung cancer with p53-negative expression had a longer 3-year survival rate than those with p53-positive expression^30^. Cox regression multivariate analysis show IL-7R, mTOR, and tumor stage were independent predictors of survival.

In conclusion, we demonstrated that IL-7 induced p53 translocation from nucleus to cytoplasm then regulating AMPK/mTOR signaling pathway to inhibit autophagy. Expression of AMPK and P53 correlate with IL-7/IL-7R level, clinical stages, and NSCLC patient survival. Together with other pro-tumorigenesis effect such as pro-proliferation, promote lymphangio -genesis, and anti-apotosis that IL-7 played in NSCLC. There still many problem to be solved. In our further study, we will investigate the interaction and balance between these cellular behavior and explore the feasibility for targeting IL-7 and combined with other inhibitor in NSCLC therapy.

## Data Availability

The data that support the findings of the present study are available from the corresponding author on reasonable request.

## Abbreviations

IL-7: Interleukin 7
AMPK: AMP-activated protein kinase
mTOR: Mammalian target of rapamycin
LC3: Light chain 3
RT-qPCR: Real-time quantitative PCR
TEM: Transmission electron microscopy
NSCLC: Non-small cell lung cancer
SCLC: Small cell lung cancer
ATG: Autophagy related gene
PFT-α: Pifithrin‐α
A-IL-7R: Anti-IL-7 receptor antibody
SDS-PAGE: SDS polyacrylamide electrophoresis
PVDF: Polyvinylidene difluoride
ECL: Electrochemiluminescence
PBS: Phosphate buffer saline
NC: Negative control
CC: Compound C
